# Descending Necrotizing Mediastinitis in an Infant

**DOI:** 10.5811/westjem.2015.1.24926

**Published:** 2015-03-02

**Authors:** Michael Butterfield, Kenshata Watkins, Enrique Palacios

**Affiliations:** * Tulane University Medical Center, Department of Internal Medicine, New Orleans, Louisiana; † Tulane University Medical Center, Department of Pediatrics, New Orleans, Louisiana; ‡ Tulane University Medical Center, Department of Radiology, New Orleans, Louisiana

## CASE

A nine-month-old girl was brought to the emergency department because of right neck swelling. She had recently been discharged from the same hospital after a brief admission for pneumonia that had followed influenza.

**Figure 1:**
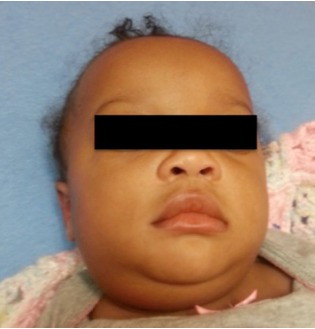
Notable swelling of the patient’s right neck.

**Figure 2:**
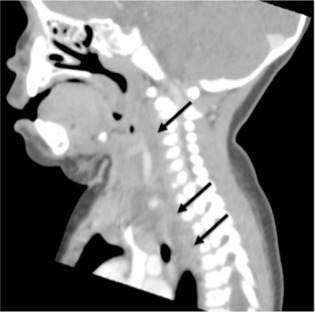
Hypointense retropharyngeal abscess tracking inferiorly into the mediastinum (arrows).

**Figure 3:**
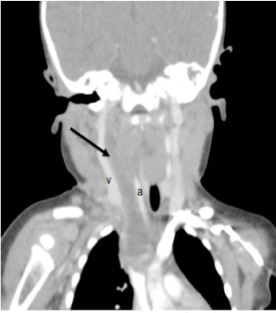
In the coronal view, the abscess (arrow) can be seen within the carotid sheath, separating the jugular vein (v) from the carotid artery (a).

The mother denied noticing increased drooling, dyspnea, or stridor. Pooled oral secretions were present on physical exam, but the patient was calm with normal vital signs (Figure 1). Computed tomography (CT) of the neck revealed a large retropharyngeal abscess tracking caudally into the posterior mediastinum (Figure 2). The infection extended into the adjacent right carotid sheath, producing a dramatic “Lincoln’s Highway” sign (Figure 3).

## DISCUSSION

Descending necrotizing mediastinitis (DNM) is a rare complication of retropharyngeal abscess (RA). The retropharyngeal space is bounded in the anteroposterior axis by the buccopharyngeal and prevertebral fascia, and extends from the base of the skull to the posterior mediastinum.^1^ Most cases of nontraumatic RA occur in children <5 years old, whose retropharyngeal lymph nodes have not yet involuted, predisposing to abscess formation.^2^,^3^

In DNM, caudal spread of the infection (by mixed flora) is facilitated by gravity and negative intrathroacic pressure.^4^ Recent reports suggest that RA and DNM are on the rise, which may be due to the increasing role of aggressive bacteria such as community-acquired methicilin-resistant staphalococcus aureus (MRSA) in their pathogenesis.^5^,^6^

Children typically present with irritability, neck pain, and increased secretions; stridor is infrequently observed. Lateral neck radiographs demonstrate widening of the prevertebral soft tissue, defined as a diameter equal or larger to that of the contiguous vertebral body.^7^ DNM is suggested by widening of the mediastinum seen on chest radiograph, but contrast-enhanced CT remains the imaging modality of choice.^8^ After airway assessment, all patients should be started on intravenous clindamycin and consulted to otolaryngology or interventional radiology to evaluate for possible abscess drainage.^3^,^9^

The patient was given one dose of IV clindamycin in the emergency department. Her airway remained patent, and she was transferred to a pediatric hospital for drainage of the abscess. She did well and was discharged home on oral antibiotics on postoperative day 5.
